# End-to-End Diverse Metasurface Design and Evaluation Using an Invertible Neural Network

**DOI:** 10.3390/nano13182561

**Published:** 2023-09-15

**Authors:** Yunxiang Wang, Ziyuan Yang, Pan Hu, Sushmit Hossain, Zerui Liu, Tse-Hsien Ou, Jiacheng Ye, Wei Wu

**Affiliations:** 1 Ming Hsieh Department of Electrical Engineering, University of Southern California, Los Angeles, CA 90089, USA; 2 The High School Affiliated to Renmin University of China, CUIWEI Campus, Beijing 100086, China

**Keywords:** deep learning, invertible neural network, metasurface, metalens, holograms

## Abstract

Employing deep learning models to design high-performance metasurfaces has garnered significant attention due to its potential benefits in terms of accuracy and efficiency. A deep learning-based metasurface design framework typically comprises a forward prediction path for predicting optical responses and a backward retrieval path for generating geometrical configurations. In the forward design path, a specific geometrical configuration corresponds to a unique optical response. However, in the inverse design path, a single performance metric can correspond to multiple potential designs. This one-to-many mapping poses a significant challenge for deep learning models and can potentially impede their performance. Although representing the inverse path as a probabilistic distribution is a widely adopted method for tackling this problem, accurately capturing the posterior distribution to encompass all potential solutions remains an ongoing challenge. Furthermore, in most pioneering works, the forward and backward paths are captured using separate models. However, the knowledge acquired from the forward path does not contribute to the training of the backward model. This separation of models adds complexity to the system and can hinder the overall efficiency and effectiveness of the design framework. Here, we utilized an invertible neural network (INN) to simultaneously model both the forward and inverse process. Unlike other frameworks, INN focuses on the forward process and implicitly captures a probabilistic model for the inverse process. Given a specific optical response, the INN enables the recovery of the complete posterior over the parameter space. This capability allows for the generation of novel designs that are not present in the training data. Through the integration of the INN with the angular spectrum method, we have developed an efficient and automated end-to-end metasurface design and evaluation framework. This novel approach eliminates the need for human intervention and significantly speeds up the design process. Utilizing this advanced framework, we have effectively designed high-efficiency metalenses and dual-polarization metasurface holograms. This approach extends beyond dielectric metasurface design, serving as a general method for modeling optical inverse design problems in diverse optical fields.

## 1. Introduction

The emergence of electromagnetic metasurfaces has sparked a transformative shift in the field of photonics research, resulting in a profound revolution in the design of optical elements [1,2,3]. These two-dimensional structures offer unprecedented control over the amplitude, phase, and polarization of incident waves, enabling tailored wavefront manipulation at subwavelength resolution. This breakthrough has facilitated the development of enormous novel elements, including beam splitters [4,5,6], polarizer [7,8,9], resonators [10,11,12], lenses [13,14,15], and holograms [16,17,18] that surpass the limitations imposed by traditional optical components. However, the widespread implementation of metasurfaces has been hindered by the challenges posed by the traditional design process. In this process, researchers manually engineer the structure and properties of the metasurface based on their knowledge and intuition, relying on trial and error with multiple iterations. While this approach has led to significant advancements, it is time-consuming and limited in its ability to explore complex design spaces and achieve optimal performance. Recent advancements in numerical algorithms have led to the emergence of inverse design methods that have revolutionized the photonic systems design process. Unlike conventional approaches, inverse design methods enable researchers to specify desired optical properties or performance characteristics, allowing algorithms to automatically generate optimal parameters to achieve those goals [19,20,21]. These inverse design methods can be broadly categorized into two pathways: optimization-based methods and deep learning-based methods. Optimization-based methods employ gradient-based algorithms like the adjoint method [22,23,24] or heuristic approaches such as genetic algorithm [25,26,27] to explore and search through the parameter space. To facilitate optimization, these methods rely on defining an objective function that quantifies the desired optical functionality, allowing the algorithms to maximize or minimize it accordingly. Nevertheless, optimization-based methods face challenges in accurately formulating objective functions, managing high computational costs, and avoiding local optima traps. In contrast, the rapid progress in artificial intelligence research has facilitated the emergence of deep learning as a promising alternative [28]. Deep learning enables the acquisition of intricate mappings between desired optical functionalities and corresponding metasurface designs, utilizing the wealth of data collected through low-cost simulations. Through training, the deep learning model becomes proficient in swiftly deducing the optimal solution for specific requirements within mere seconds, diminishing computational complexity and enabling rapid design iterations. Moreover, it possesses the remarkable ability to generate novel designs that may not have been present in the training data.

Discriminative deep learning models, such as multilayer perceptrons (MLPs) [29,30,31], convolutional neural networks (CNNs) [32,33,34], and recurrent neural networks (RNNs) [35,36,37], were initially introduced in the domain of metasurface design problems. Their primary objective is to offer a single geometric parameter prediction for each optical response input. However, the inherent complexity of the metasurface design space, wherein multiple designs can yield the same optical response, poses challenges for these models [38]. In contrast, generative models possess the capability to model the mapping between geometric parameters and optical responses as a probability distribution, enabling them to naturally accommodate one-to-many mappings. Moreover, generative models can uncover optimal new designs that may not even exist within the dataset. Various generative models, including generative adversarial networks (GANs) [39,40,41] and variational autoencoders (VAEs) [42,43,44], have been adopted in different inverse design frameworks. However, both GANs and VAEs face difficulties in directly estimating likelihood, which poses a challenge when evaluating the quality of generated samples and undermines their generating performance. To address these limitations, normalizing flows (NFs) have emerged as a promising approach. NFs focus on modeling complex distributions by transforming a simple base distribution using a series of invertible transformations [45,46,47]. This approach provides an explicit and tractable likelihood estimation, overcoming the likelihood estimation challenges faced by GANs and VAEs. With NFs, it becomes possible to calculate the probability density of a given sample, which is essential for accurately modeling complex distributions. Furthermore, NFs can efficiently model one-to-many mappings, which is crucial for capturing multiple designs that can yield the same optical response in metasurface design problems. This capability allows NFs to effectively address the challenge of designing metasurfaces with diverse geometrical configurations that produce identical optical responses.

In this study, we presented an inverse design framework based on NFs to efficiently design dielectric metasurfaces. Our approach leverages NFs to construct an invertible neural network (INN) [48] capable of simultaneously modeling the forward simulation process and the inverse design process. Unlike most deep learning-based methods that require separate models for forward and inverse tasks, the inherent invertibility of NFs enables a single INN to handle both, leading to a significant reduction in system complexity and computational resources. INNs have consistently demonstrated their ability to accurately model probability distributions across various inverse problems compared to GAN and VAE, resulting in predictions characterized by a high degree of diversity [49]. To demonstrate the effectiveness of our methodology, we integrated the INN with the angular spectrum method, resulting in an end-to-end metasurface design and evaluation framework that is both efficient and accurate. We applied this framework to two design tasks: Firstly, we employed it to design a metalens based on rectangular dielectric meta-atoms. The simplicity of the structure allowed us to effectively present the INN’s precise modeling capabilities for both forward prediction and inverse retrieval. Secondly, we employed it to design dual-polarization metasurface holograms based on complex dielectric meta-atoms. These meta-atoms offered full control over both amplitude and phase, enabling us to demonstrate the successful design of intricate holograms. Through this task, we validated the capability of our model to handle complex design challenges effectively. Our proposed framework offers easy extensibility to various other design tasks, thereby stimulating a continuous wave of applying deep learning techniques to revolutionize the field of optical design.

## 2. Materials and Methods

### 2.1. Problem Description

To provide a clear description of the problem, we adopt the notation $x\in R^{m}$ to denote the design parameters and $y\in R^{n}$ to represent the target response. In our case, x is a vector that captures the geometric shape of a metasurface while y is also a vector that describes the transmission coefficients. In the forward process, our objective is to find an accurate target y for a given design parameter x, which can be expressed as y=f(x). This mapping is often well-defined and tractable. In contrast, the inverse process aims to find a design parameter x that is the best fit for a given target y. Due to the fact that one target can correspond to many design parameters, it is natural to express the inverse design process as a conditional probability, $p\left(x|y\right)$. This probability represents the likelihood of obtaining a particular design parameter x given a specific target y. To approximate the full posterior distribution $p\left(x|y\right)$ using a tractable model, we incorporate a latent random variable $z\in R^{d}$, which is drawn from a multi-variate standard normal distribution. This allows us to reparametrize the posterior distribution $p\left(x|y\right)$ as a deterministic function g of y and z. If we apply an invertible network with parameter θ to implement both the forward function f and the inverse function g, the forward and backward process can be written as:

(1) $$\left[y,\;z\right]=f\left(x;\theta \right)$$

(2) $$x=g\left(y,\;z;\theta \right);z\sim N\left(0,I^{d}\right)$$

**Figure 1:**
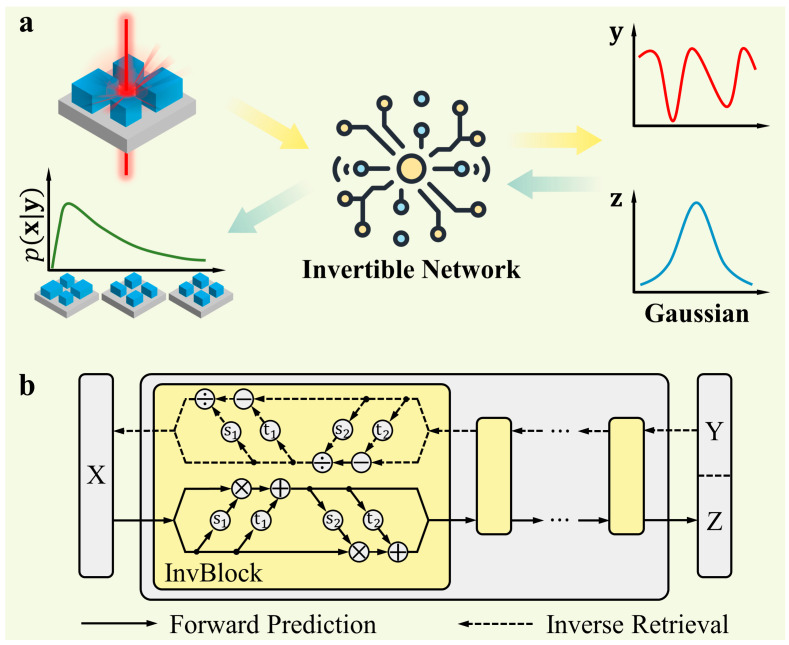
INN for metasurface design. (**a**) The INN serves a dual role by modeling both forward prediction and inverse retrieval. During forward prediction, the network takes an input design parameter **x** and generates a specific target **y**, along with a vector **z** that conforms to a Gaussian distribution. In the case of inverse retrieval, the network utilizes a target **y** and a vector **z** drawn from a Gaussian distribution as inputs. This allows the model to predict the corresponding design parameter **x**. (**b**) The structure of INN. It consists of multiple invertible blocks. Each block applies a simple transformation from the input to the output. By chaining these blocks together, a complex transformation from the input to the output can be achieved.

Figure 1a illustrates these two processes. Since $f=g^{-1}$ is enforced by the invertible network, the dimension on both the input and output sides should match. When m>n, z must have dimension d=m−n. When m<n, we can append x with a zero vector $x_{0}\in R^{n-m+d}$.

### 2.2. Network Architecture

We have adopted the INN architecture proposed by Ardizzone et al. [48]. As shown in Figure 1b, the primary component of this network is an invertible block that consists of two complementary affine coupling layers. The equations defining an invertible block, taking the input $x=[x_{1},x_{2}]$ and producing the output $y=[y_{1},y_{2}]$, are as follows:

(3) $$y_{1}=x_{1}⊙\exp\left(s_{1}\left(x_{2}\right)\right)+t_{1}\left(x_{2}\right)$$

(4) $$y_{2}=x_{2}⊙\exp\left(s_{2}\left(y_{1}\right)\right)+t_{2}\left(y_{1}\right)$$

These two equations can be easily inverted:

(5) $$x_{1}=\left(y_{1}-t_{1}\left(x_{2}\right)\right)⊙\exp\left(-s_{1}\left(x_{2}\right)\right)$$

(6) $$x_{2}=\left(y_{2}-t_{2}\left(y_{1}\right)\right)⊙\exp\left(-s_{2}\left(y_{1}\right)\right)$$

It is important to note that the mapping functions $s_{i}$ and $t_{i}$ can be arbitrarily complex and do not need to be invertible. Therefore, they can be implemented using neural networks to achieve intricate transformations between the input and output. The INN is constructed by connecting multiple of these invertible blocks in a sequence to achieve a complex transformation between the geometry parameters and the transmission coefficients. The neural network models were constructed under the open-source deep learning framework of PyTorch. More details about the INN structure and training can be found in Supplementary Materials Section S1.

### 2.3. Loss Definition

By leveraging the invertibility of the model, it becomes possible to define the loss function on both the input and output sides. For the forward prediction, we need to define the loss function for both y and z. For y, we penalize deviations between the network predictions and the ground truth simulation results with an MSE loss $L_{y}=\frac{1}{n}{\sum_{i}\left(y_{i}-\hat{y}\right)}^{2}$, where $\hat{y}$ is the ground truth. On the other hand, for z*,* we aim to enforce two essential aspects: firstly, the generated z must follow the standard normal distribution N(0, I) and secondly, z should not have impact on y, meaning they should be independent to each other. Hence, we seek to achieve $q\left(f_{y}(x),f_{z}(x)\right)=q\left(f_{y}\left(x\right)|f_{z}\left(x\right)\right)q\left(f_{z}\left(x\right)\right)=p\left(y\right)N\left(0,\;I\right)$. Here, $q\left(f_{y}(x),f_{z}(x)\right)$ represents the joint distribution of the forward prediction and $p\left(y\right)$ represents the distribution of the ground truth. However, since we only have access to data rather than the distribution itself, we require a method to evaluate the distribution when only data are available. To address this, we utilize Maximum Mean Discrepancy (MMD), a kernel-based method that can compare two probability distributions using samples. The detail about MMD is introduced in Supplementary Materials Section S2. By applying MMD, we can assess the dissimilarity between the generated distribution $q\left(f_{y}(x),f_{z}(x)\right)$ and the desired distribution $p\left(y\right)N\left(0,\;I\right)$. The loss for z is defined as $L_{z}=MMD\left(q\left(f_{y}\left(x\right),\;f_{z}\left(x\right)\right),p\left(y\right)N\left(0,\;I\right)\right)$. To ensure the model accurately captures the posterior $p\left(x|y\right)$, we introduce an additional loss term on the input side. This loss, denoted as $L_{x}$ and computed using MMD, compares the distribution $p\left(x\right)$ representing the ground truth x with the distribution $q\left(g\left(y,z\right)\right),$ representing the model’s prediction. By incorporating $L_{x}=MMD\left(p\left(x\right),\;q\left(g\left(y,z\right)\right)\right)$ into the training process, we aim to encourage the model to produce predictions that align closely with the true distribution $p\left(x\right)$. In summary, the total loss can be written as:

$$L=\lambda_{1}L_{x}+\lambda_{2}L_{y}+\lambda_{3}L_{z}$$

where $\lambda_{1}$, $\lambda_{2},$ and $\lambda_{3}$ are hyperparameters that control the weight assigned to each loss term.

### 2.4. Simulation Setup

The training data were generated by systematically sampling the design space and subsequently calculating the corresponding transmission coefficients. These sampled designs were input into the commercial simulation software Ansys Lumerical FDTD (2023 R1) to obtain the corresponding transmission coefficients. Within the simulation setup, the silicon dioxide (SiO_2_) substrate was represented as a lossless dielectric with a refractive index set to 1.45. The refractive index of titanium dioxide (TiO_2_) was obtained from the database within Ansys Lumerical FDTD [50]. For boundary conditions, periodic boundary conditions were applied in the x- and y-directions, while the Perfect Matching Layer (PML) was utilized as the boundary in the z-direction. In the simulation, the source was positioned 150 nm below the meta-atom and the field monitor was set 500 nm above the meta-atom. It is worth noting that as the fields propagate from the source to the meta-atom and subsequently to the monitors, they accumulate additional phases. However, this additional phase was compensated for, ensuring that only the phase delay introduced by the meta-atom itself was taken into consideration.

## 3. Results and Discussion

### 3.1. Inverse Design of Metalens

**Figure 2:**
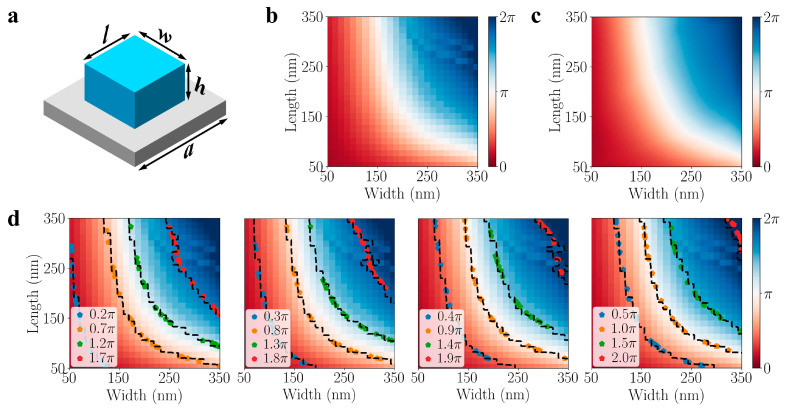
(**a**) The parameters that describe the nanopost unit cell. (**b**) The simulated phase delay mapping. (**c**) The phase delay mapping predicted by the INN. (**d**) The predicted design parameters for different targets. The dashed lines represent the contours for each target in the ground truth while the pentagon dots represent the predicted parameters for a given target.

To thoroughly evaluate the model’s capacity to handle both the forward and inverse design processes, we initially utilized it to tackle a well-defined problem: the design of metalens based on rectangular dielectric meta-atoms. As shown in Figure 2a, the unit cell consists of a dielectric nanopost placed on a SiO_2_ substrate, characterized by geometry parameters including the lattice constant (*a*) and the dimensions of the nanopost, specifically the width (*w*), length (*l*), and height (*h*). In this particular case, we aimed for the metalens to function at a wavelength of 580 nm for x-polarization. To achieve this, we utilized TiO_2_ as the material for the nanopost as it possesses a high refractive index and exhibits low loss at this specific wavelength. The height of the nanopost was set at a fixed value of 600 nm while the periods along both the x- and y-directions were maintained at 400 nm. Consequently, there were only two design parameters that needed consideration: the width (*w*) and length (*l*) of the nanocube. The limited number of parameters makes it convenient for us to visualize the outcomes of both the forward and inverse processes. To cover the entire 2π phase range while preserving a transmission amplitude close to 1, these two design parameters were independently sampled from a uniform distribution ranging from 50 nm to 350 nm. In total, a dataset of 10,000 training examples was generated through FDTD simulations. This dataset was subsequently split into a training dataset (90%) and a testing dataset (10%). The amplitude of the complex transmission coefficient is displayed in Figure S4 in the Supplementary Materials. Given that the magnitude of the complex transmission coefficient for all the meta-atoms in our dataset exceeds 0.93, our analysis in this context focuses exclusively on the phase delay as the target parameter. Figure 2b illustrates the simulated results of the phase delay across the whole width and length range. It is apparent that multiple width and length configurations can yield the same phase delay. This observation highlights the challenging nature of the inverse process as it entails mapping the phase to different design parameters across a broad range. Despite being a relatively simple design problem, it exhibits a significant one-to-many relationship, where one phase delay can map to multiple design parameters. This characteristic can pose difficulties for many deep learning models, making it a non-trivial task to accurately predict the appropriate width and length given a specific phase delay. However, in this study, we will demonstrate the effectiveness of our INN model in accurately predicting outcomes for both forward prediction and inverse retrieval.

To begin with, we evaluated the accuracy of the INN model for the forward process. We sampled the width and length, with a 3 nm increment, within the range of 50 nm to 350 nm, resulting in a total of 10,201 evaluation points. Using our trained INN model, we predicted the phase delay for each configuration and the corresponding results are shown in Figure 2c. Comparing these predictions to the ground truth depicted in Figure 2b, we observe highly precise estimations of the phase delay across the entire spectrum of design parameters. The average prediction mean squared error (MSE) is calculated to be 0.006, indicating the model’s strong performance in capturing the desired outcomes. In the context of the inverse process, our expectation is that our model will effectively capture the entire posterior distribution. Consequently, it should be capable of generating a broad spectrum of design parameter combinations within the feasible region, displaying significant diversity while maintaining a consistent target phase delay. To validate this, we selected 16 different targets in the 2π phase range. For each target, we employed the INN model to predict 20 samples. The results are presented in Figure 2d, where the dashed lines depict the contour lines of each target phase in the ground truth and the points represent the predicted results for each target. Upon observation, we can clearly see that all the predicted points closely align with their corresponding contour lines, indicating accurate predictions. Additionally, it is evident that the generated parameters span the entire feasible region, illustrating the model’s ability to capture the entire posterior and produce diverse design parameters.

**Figure 3:**
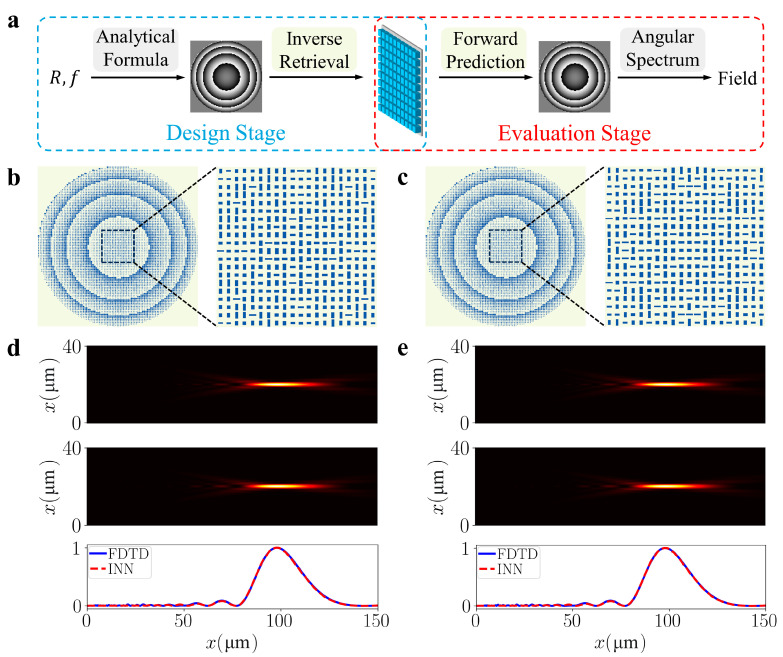
End-to-end design and evaluation framework for dielectric metalens. (**a**) Flowchart of the design stage (in blue box) and the evaluation stage (in red box). (**b**,**c**) Two inverse-designed results. (**d**,**e**) The predicted and simulated field distributions for the two metalenses are illustrated in the figures. In each figure, the top image corresponds to the result predicted by our framework while the middle image represents the result calculated by FDTD simulation. The bottom image displays the intensity profiles along the cutline at x=20 μm for both methods.

Due to the accurate predictions of the INN model for both design parameters and optical responses, we can achieve an end-to-end design and evaluation framework for dielectric metasurfaces by integrating the INN model with the angular spectrum method. This approach effectively eliminates the need for any empirical human intervention and significantly expedites the design process. The framework for designing dielectric metalenses is shown in Figure 3a. In the design stage, the required wavelength, lens radius, and focal length are utilized to obtain the necessary phase map through an analytical formula: $\varphi \left(x,\;y\right)=\frac{2\pi }{\lambda }(f-\sqrt{f^{2}+x^{2}+y^{2}})$. Following this, the INN model is employed to retrieve the physical meta-atom designs by taking the phase requirement at each pixel as the input. During the evaluation stage, the forward path of the INN is leveraged to predict the actual phase map produced by the metasurface. Subsequently, the electric field profiles along the propagation direction are generated by propagating light from the metasurface plane. We used the angular spectrum method to model the propagation of a wave field, which is a highly efficient method. To demonstrate the effectiveness of our framework, a metalens was designed with a 20 µm radius and a 100 µm focal length, operating at 580 nm. Figure 3b,c depicts two designed metalens examples, demonstrating the capability of our framework to generate diverse metasurfaces for a given target due to the INN’s ability to capture the one-to-many mapping in the retrieval process. During the evaluation stage, the INN model utilizes the generated designs as an input to predict their performance. Additionally, we perform FDTD simulations to verify the generated designs. The results for both designs are presented in Figure 3d,e. It is evident that our predicted results align closely with the simulated results, validating the accuracy of both the forward prediction and inverse retrieval processes. Furthermore, our framework exhibits remarkable efficiency, completing the entire process in less than 10 s, regardless of the complexity of the structure. In comparison, performing FDTD simulations alone can take over 10 min in this example.

### 3.2. Inverse Design of Dual-Polarization Metasurface Holograms

**Figure 4:**
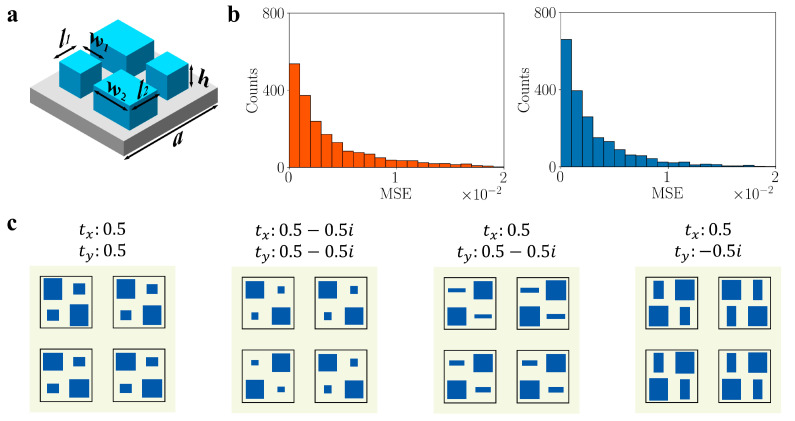
(**a**) The parameters that describe the complex meta-atom. (**b**) Histogram distribution of the forward prediction for $t_{x}$ (**left**) and $t_{y}$ (**right**) in the testing dataset. (**c**) The top four inverse retrieved meta-atom designs for four different transmission coefficient targets. The summary of the predicted and simulated performance of these meta-atoms is in Supplementary Materials Section S3.

In the previous section, we showcased the INN model’s ability to accurately model both the forward prediction and inverse retrieval simultaneously. However, considering the limited control offered by the simple geometry of the nanopost, we will now address a more complex problem. Specifically, we will employ the INN model to tackle the design of dual-polarization complex-amplitude metasurface holograms. In contrast to phase-only modulated holograms, complex-amplitude holograms enable faithful reconstruction of high-quality images by modulating both the amplitude and phase of the incident field [51]. To achieve full control over the amplitude and phase for both x- and y-polarizations, a straightforward design strategy involves integrating multiple nanostructures within a single unit cell to construct a complex meta-atom [52,53,54]. However, the inherent complexity of the near-field coupling effect has led most conventional methods to address it by maintaining a significant separation between each nanostructure. This decoupling approach aims to mitigate the interaction between individual nanostructures and simplify the design process but at the expense of significantly increasing the size of the device. In contrast, our approach benefits from the powerful INN model, which can model the full posterior of the inverse retrieval. As a result, we can obtain the design parameters for any complex transmission coefficient without relying on any decoupling efforts. Figure 4a illustrates the schematic of the complex meta-atom. The meta-atom is positioned on a SiO_2_ substrate and comprises four identical square subcells, with each subcell containing a rectangular TiO_2_ nanopillar situated at its center. The lattice constant (*a*) for the meta-atom is 720 nm and the height (*h*) of each nanopillar is 600 nm. To ensure that the meta-atom achieves consistent performance for both x and y polarization, the two pillars located on each diagonal are identical in design. This particular configuration results in four design parameters, namely *w*_1_, *l*_1_, *w*_2_, and *l*_2_, which correspond to the sizes of the two distinct nanopillars within the meta-atom. As a result of the polarization-dependent near-field coupling between the nanopillars, the meta-atom can attain comprehensive control over both phase and amplitude. A pioneering study has explored the utilization of a similar meta-atom configuration for constructing transmissive metasurfaces operating in the terahertz range [55]. The researchers relied on extensive FDTD simulation data to manually select a specific meta-atom configuration that fulfilled a given amplitude and phase requirement, establishing a one-to-one mapping between the geometry parameters and the transmission coefficients. However, this manual approach has limitations, as certain designs that could potentially achieve higher agreement for specific transmission coefficients may not be included in the collected data and could be overlooked during the design process. In this section, we will demonstrate the efficiency of our INN-based automatic design and evaluation framework for designing metasurface holograms.

Our objective is to design a metasurface hologram operating at 633 nm for both x- and y-polarization. To accomplish this, we randomly sample the four design parameters within a range of 50 nm to 300 nm. Subsequently, we calculate the complex transmission coefficient for both x- and y-polarizations at 633 nm for each meta-atom using FDTD simulation. In total, we collected 50,000 labeled data points, utilizing 48,000 for training and 2000 for testing. Figure 4b illustrates the histogram distribution of mean squared errors for the predicted $t_{x}$ and $t_{y}$ in the testing dataset. The long-tailed distribution clearly demonstrates the accuracy of the forward prediction as the majority of errors fall within 0.04. For the inverse retrieval task, we expect the model to generate diverse meta-atom designs for a given transmission coefficient target. However, due to the near-field coupling effect inside the meta-atom unit cell, the posterior for a given transmission coefficient can be quite complex. Although the model attempts to model the full posterior, not all generated designs exhibit equally high quality. To address this, we employ the forward prediction model to reevaluate all generated designs and sort them based on the distance between the predicted coefficients and the target coefficients. We then select the top few designs as the final candidates. Figure 4c illustrates the top four designs for four different transmission coefficient targets, showcasing the variation in the designed results. Importantly, none of these generated designs exist in the training data, showcasing the model’s ability to explore novel and diverse solutions. To further validate our results, we analyzed the real transmission coefficient for each design using FDTD simulation. The detailed findings are summarized in Supplementary Materials Section S3, revealing good agreement between the predicted results and the simulation outcomes.

**Figure 5:**
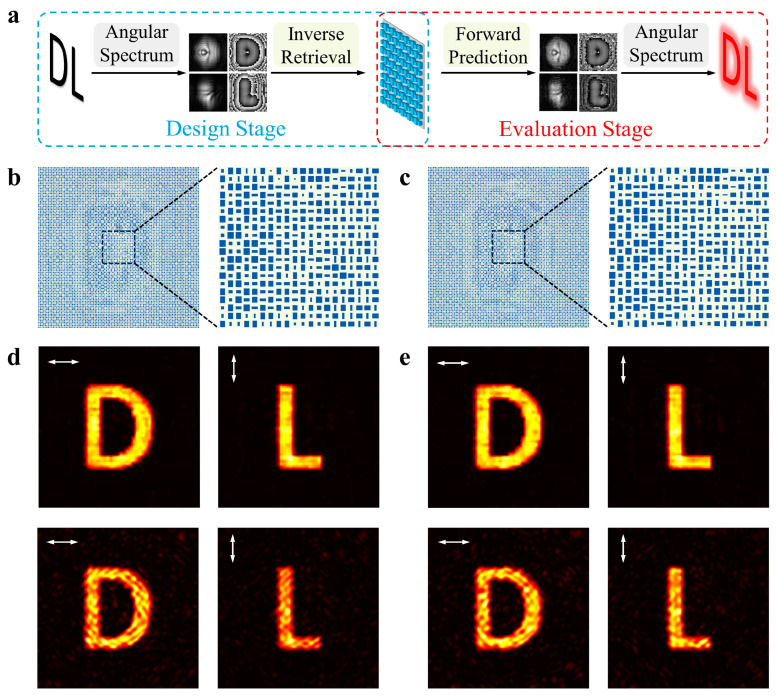
End-to-end design and evaluation framework for dual-polarization metasurface holograms. (**a**) Flowchart of the design stage (in blue box) and the evaluation stage (in red box). (**b**,**c**) Two inverse-designed results. (**d**,**e**) The predicted and simulated field distribution for the two holograms. The arrows indicate the polarization of the incident filed. In each figure, the top image corresponds to the result predicted by our framework and the bottom image represents the result calculated by FDTD simulation.

As discussed in the preceding section, the INN can be integrated with the angular spectrum method to realize an end-to-end design and evaluation framework. As depicted in Figure 5a, in the design stage, the required phase and amplitude map for both polarizations are retrieved by reversing the propagation of the light from the virtual object on the imaging plane to the metasurface plane. Then, the phase and amplitude requirement at each pixel is fed to the INN model to retrieve the physical meta-atom designs. The complete metasurface is derived by combining all meta-atoms according to their position coordinates. In the evaluation stage, the actual phase and amplitude map produced by the metasurface is predicted using the forward path of the INN. Subsequently, the hologram images on the image plane are generated by propagating the light from the metasurface plane. To demonstrate the effectiveness of our proposed framework, we have chosen the letters “D” and “L” as the target images for x- and y-polarization, respectively, positioned at 50 μm from the metasurface. The metasurface encoding these two letters measures 36 μm × 36 μm in size and contains 50 × 50 meta-atoms. In Figure 5b,c, we illustrate two metasurfaces from the design stage and provide a zoom-in view for the center 10 × 10 meta-atoms. It is evident that these two metasurfaces contain completely different meta-atom designs for some pixels. Moving on, both metasurface designs are then processed through the evaluation stage to generate holograms for both x- and y-polarization, as shown at the top in Figure 5d,e. Remarkably, both metasurfaces can generate clear target images with a high signal-to-noise ratio. To further validate our results, we input both designed metasurfaces into FDTD simulation software (Ansys Lumerical FDTD 2023 R1) to obtain the hologram images. The simulated results, depicted at the bottom in Figure 5d,e, exhibit good agreement with the predicted images by our framework, thus confirming the capability of our proposed design method. Our framework is noteworthy for its complete elimination of human intervention, enabling the design and evaluation process to be accomplished in just 10 s. In contrast, the FDTD simulation alone requires approximately 2 h to yield the simulation result.

## 4. Conclusions

We have successfully demonstrated the effectiveness of the INN model in automating the design process of dielectric metasurfaces. Unlike other deep learning models that require separate models for forward prediction of optical properties and backward retrieval of geometry parameters, the INN’s unique invertibility allows it to simultaneously model both processes, significantly simplifying the inverse design system. By incorporating an additional latent variable and defining training loss for both forward and backward predictions, the INN accurately predicts the optical properties of a structure and captures the complete posterior distribution of the geometry parameters associated with a specific optical target. This capability empowers the model to generate a wide range of structures that meet the desired requirements. Through the seamless integration of the INN model with the lens phase transformation equation and the angular momentum method, we have established an end-to-end metalens design and evaluation framework that covers the entire design process. Additionally, our success in realizing complex amplitude holograms with various configurations highlights the impressive capabilities of the INN model in metasurface design. Unlike most conventional design methods that attempt to decouple each nanostucture within the unit cell, our model considers the entire complex meta-atom and models the full posterior for reverse retrieval. As a result, our approach can generate new designs that were not present in the training data, significantly expanding the design space and facilitating the exploration of novel solutions. Importantly, INN does not impose constraints on the dimension of the input and output. Therefore, this approach can be extended to design broadband or multi-band metasurfaces. This versatility goes beyond the design of parametric metasurfaces typically characterized by a limited number of parameters and can encompass the creation of free-form metasurfaces. Furthermore, it is not limited solely to dielectric metasurfaces but can serve as a versatile automatic design tool for a wide range of optical design tasks. By automating the design process, it enables faster iterations, reduces the reliance on expert knowledge, and facilitates rapid prototyping and the deployment of customized optical devices.

## Data Availability

The data that support the findings of this study are available from the corresponding author upon reasonable request.

## Supplementary Materials

The following supporting information can be downloaded at: https://www.mdpi.com/article/10.3390/nano13182561/s1, Figure S1. Structure of $s_{i}$ and $t_{i}$ in the INN for designing (a) metalens and (b) holograms. Table S1. Network structures of InvBlock. Figure S2. The training loss of INNs for designing (a) metalens and (b) holograms. Figure S3. Maximum Mean Discrepancy (MMD) analysis of two-dimensional normal distributions with different mean and standard variation. The MMD values for (a) and (b) are 0.509 and 0.063 respectively. Figure S4. The amplitude of the complex transmission coefficient of the nanopost. Table S2. Prediction and re-simulation result for inverse-designed meta-atom. Figure S5. Comparison between (a) and (c) complex amplitude and (b) and (d) phase-only metasurface holography. Figure S6. The inverse designed metalens using INN trained with (a) 9000 data points and (b) 7000 data points. Refs. [48,56,57] are cited in the Supplementary Materials.
