# Supplementary material for: End-to-End Diverse Metasurface Design and Evaluation Using an Invertible Neural Network
Source: Nanomaterials (Basel). 2023 Sep 15;13(18):2561. doi: 10.3390/nano13182561 (PMC10534592; doi:10.3390/nano13182561)
Supplement: Supplementary file 1 [file nanomaterials-13-02561-s001.zip › nanomaterials-2572226-supplementary.pdf]

nanomaterials

MDPI

Supplementary Materials

End-to-End Diverse Metasurface Design and Evaluation Using an Invertible Neural Network

Yunxiang Wang [1], Ziyuan Yang [2], Pan Hu [1], Sushmit Hossain [1], Zerui Liu [1], Tse-Hsien Ou [1], Jiacheng Ye [1] and Wei Wu [1,*]

[1] Ming Hsieh Department of Electrical Engineering, University of Southern California, Los Angeles, CA 90089, USA
[2] The High School Affiliated to Renmin University of China, CUIWEI Campus, Beijing 100086, China
* Correspondence: wu.w@usc.edu

S1. The Architecture of INN

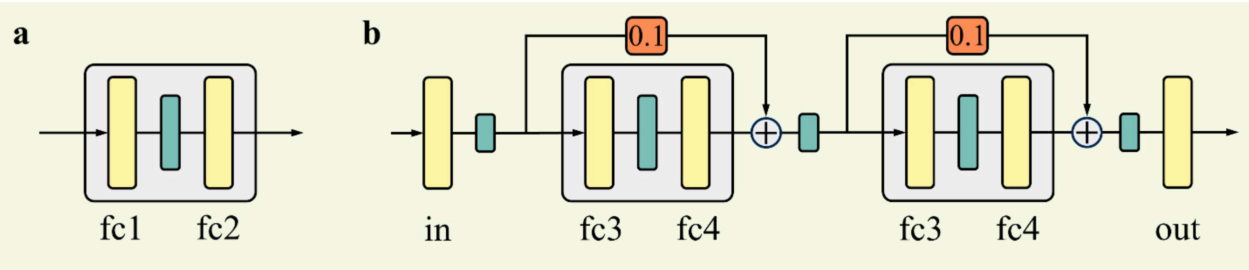

Figure S1. Structure of s_i and t_i in the INN for designing a) metalens and b) holograms.

Figure S1 depicts the neural network architectures of s_i and t_i within the InvBlock, utilized in the design of metalens and metasurface holograms respectively. For metalens design, the network consists of a simple 2-layer fully connected structure. In contrast, for hologram designs, the network incorporates two ResBlocks, each comprising an input mapping and an output mapping. We adopt residual rescaling[1] with a rescaling factor set to 0.1 to ensure the stability of the training process. For metalens design, the geometry parameters (x) have a dimension of 2, the optical response (y) has a dimension of 1, and the latent variable (z) has a dimension of 2. To enhance the network's capability to learn complex transformations, we follow previous recommendations[2] and pad both the input and output with zeros, extending them to a length of 12. In this case, the INN comprises 3 InvBlocks. Table 1 summarizes the network structures for both design tasks. In the base cases, the LeakyReLU activation function is utilized. The Adams optimizer is employed for optimization, initialized with a learning rate of 0.001. During training, the learning rate is exponentially decayed with a factor of 0.97. Specifically, the network used for metasurface design was trained for 100 epochs, while the network used for designing holograms underwent training for 200 epochs.

Table S1. Network structures of InvBlock.

Task	Layers	Input Dimension	Output Dimension
Metalens Design	fc1	12	64
	fc2	64	12
Holograms Design	in	12	64
	fc3	64	64
	fc4	64	64
	out	64	12

The training times for the two models were as follows: the first model required 8 minutes for training, while the second model took 18 minutes to complete training. Both of these models were trained on a single RTX 3090 GPU. Figure S2 provides an illustration of the training loss for the INN applied to both metalens and hologram designs. In the case of the INN used for designing metalenses, the values of λ_1, λ_2 and λ_3 in equation (1) from the main manuscript were set to 200, 50, and 100. For the INN used for designing holograms, the values of λ_1, λ_2 and λ_3 were adjusted to 250, 50 and 100.

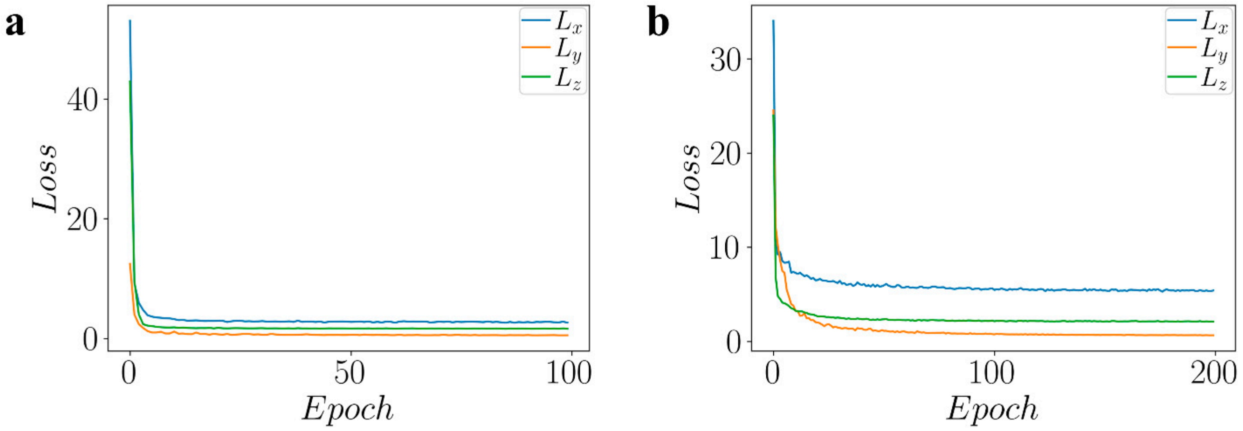

Figure S2. The training loss of INNs for designing a) metalens and b) holograms.

S2. Maximum Mean Discrepancy

Maximum mean discrepancy (MMD) is a kernel-based method employed to measure the dissimilarity or discrepancy between two probability distributions.[3]. It is based on the concept that if two distributions are identical, their expected values should be similar across all functions within a specific function class. MMD quantifies this discrepancy by comparing the expected values of functions evaluated on samples from the two distributions. Due to its ability to capture distributional differences, MMD is often utilized as a loss function in a variety of machine learning algorithms.

Let's begin by introducing the definitions of a kernel and a reproducing kernel Hilbert space (RKHS). A kernel, denoted as k, is a positive definite function $\chi \times \chi \rightarrow \mathbb{R}$, satisfying the property that for any $n \in \mathbb{N}$ and real numbers $c_1, \dots, c_n \in \mathbb{R}$, and any elements $x_1, \dots, x_n \in \chi$.

$$\sum_{i=1}^{n} \sum_{j=1}^{n} a_i a_j k(x_i, x_j) \geq 0$$

An RKHS, denoted as $\mathcal{H}$, associated with a kernel k, is a space of functions spanned by functions $k(x,\cdot)$ for all $x \in \chi$. In this space, the inner product is defined by:

$$\langle k(x_1,\cdot), k(x_2,\cdot) \rangle = k(x_1, x_2)$$

This property is known as the kernel trick, which enables us to work with implicit feature spaces without explicitly evaluating the feature map.

Now, let's introduce the concept of mean embedding. Given a probability distribution P for a random variable X, the mean embedding is another feature map that takes $\phi(X)$ and maps it to the means of every coordinate of $\phi(X)$:

$$\mu_p(\phi(X)) = \left[E(\phi(X_1)), \cdots, E(\phi(X_m))\right]^T$$

Inner product of the mean embeddings of $X \sim P$ and $Y \sim Q$ can be written in terms of kernel function such that:

$$\langle \mu_P(\phi(X)), \mu_Q(\phi(Y)) \rangle = E_{P,Q}[\langle \phi(X), \phi(Y) \rangle] = E_{P,Q}[k(X, Y)]$$

Given two random variables X and Y, the maximum mean discrepancy is the distance between feature means of X, Y:

$$\begin{aligned}\mathrm{MMD}^2(\mathrm{P},\mathrm{Q}) &= \left\|\mu_P-\mu_Q\right\|_{\mathcal{F}}^2\\ &= \langle\mu_P-\mu_Q,\mu_P-\mu_Q\rangle\\ &= \langle\mu_P,\mu_P\rangle-2\langle\mu_P,\mu_Q\rangle+\langle\mu_Q,\mu_Q\rangle\\ &= E_P[k(X,X)]-2E_{P,Q}[k(X,Y)]+E_Q[k(Y,Y)]\end{aligned}$$

This expression quantifies the dissimilarity or discrepancy between the feature means of the two random variables. By calculating the MMD, we can assess the divergence between the distributions represented by X and Y based on their feature means. In real life settings, we don't have access to the underlying distribution of data. For this reason, it is possible to use an estimate for the equation:

$$\mathrm{MMD}^2(X,Y)=\frac{1}{m(m-1)}\sum_i\sum_{j\neq i}k(\mathbf{x_i},\mathbf{x_j})-2\frac{1}{mm}\sum_i\sum_j k(\mathbf{x_i},\mathbf{y_j})+\frac{1}{m(m-1)}\sum_i\sum_{j\neq i}k(\mathbf{y_i},\mathbf{y_j})$$

Figure S2 illustrates an example of Maximum Mean Discrepancy (MMD) analysis. In both scenarios, 100 samples are randomly drawn from each distribution, and the MMD is calculated using a Gaussian kernel. In Figure S2a), the set of samples denoted as X is drawn from a two-dimensional normal distribution with mean vector $\boldsymbol{\mu}=[0,0]^T$ and $\boldsymbol{\Sigma}=\begin{bmatrix}1&0\\0&1\end{bmatrix}$. The set of samples denoted as Y is drawn from another two-dimensional normal distribution with mean vector $\boldsymbol{\mu}=[1,1]^T$and $\boldsymbol{\Sigma}=\begin{bmatrix}2&-1\\-1&2\end{bmatrix}$. The calculated MMD for this scenario is 0.509. In Figure S2b, both sets of samples, X and Y, are drawn from the same normal distribution with mean vector $\boldsymbol{\mu}=[0,0]^T$and $\boldsymbol{\Sigma}=\begin{bmatrix}1&0\\0&1\end{bmatrix}$. The calculated MMD for this scenario is 0.063. In this work, we followed the suggestion to use the Inverse Multiquadratic kernel $k(x,y)=\frac{a^2}{a^2+\|x-y\|_2^2}$ with multiple bandwidths a. Compared to the gaussian kernel, the Inverse Multiquadratic kernels provide heavier tails than Gaussian that needed to get meaningful gradients for outliers.

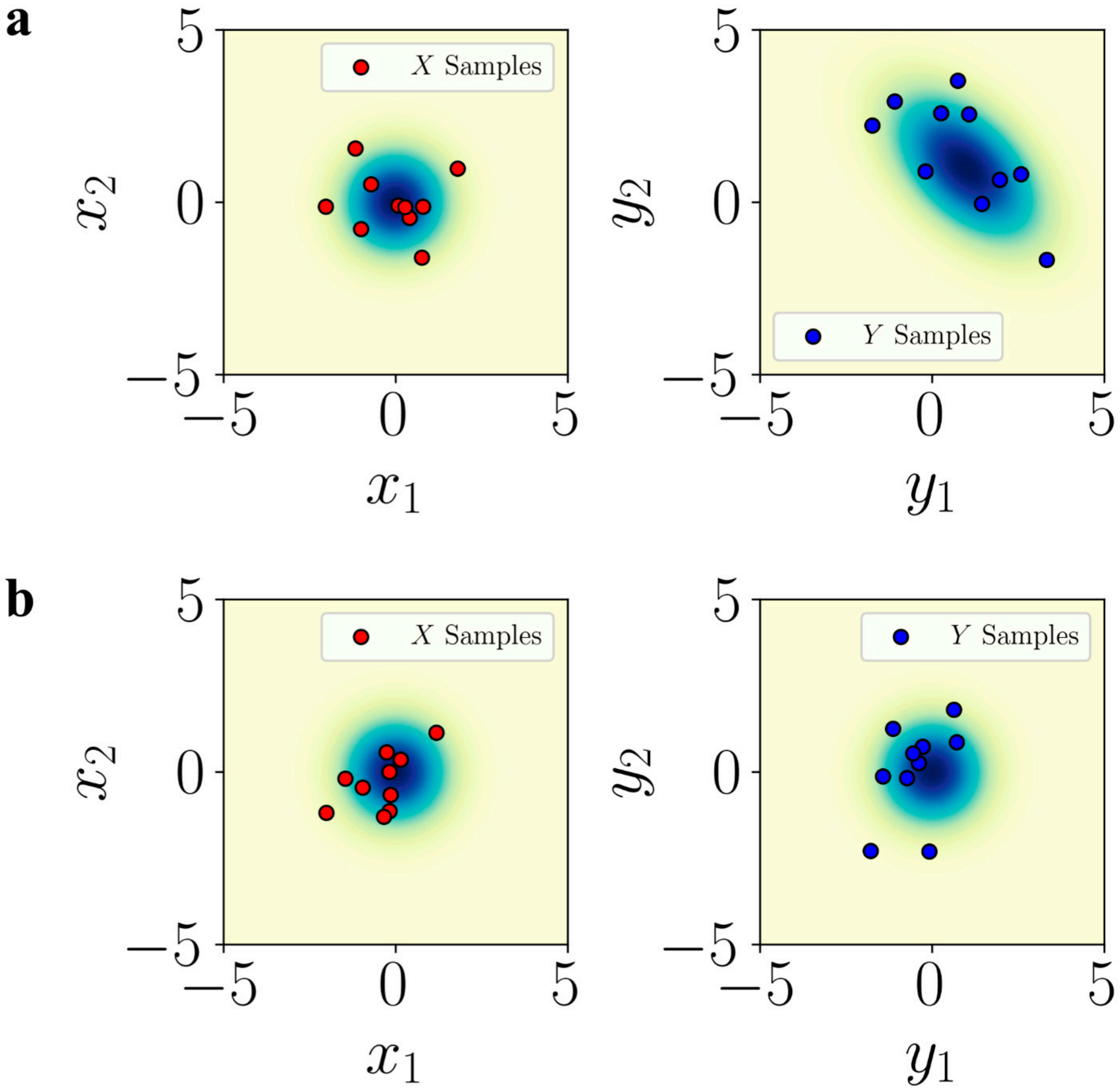

Figure S3. Maximum Mean Discrepancy (MMD) analysis of two-dimensional normal distributions with different mean and standard variation. The MMD values for a) and b) are 0.509 and 0.063 respectively.

S3. The Amplitude of the Transmission of Nanopost

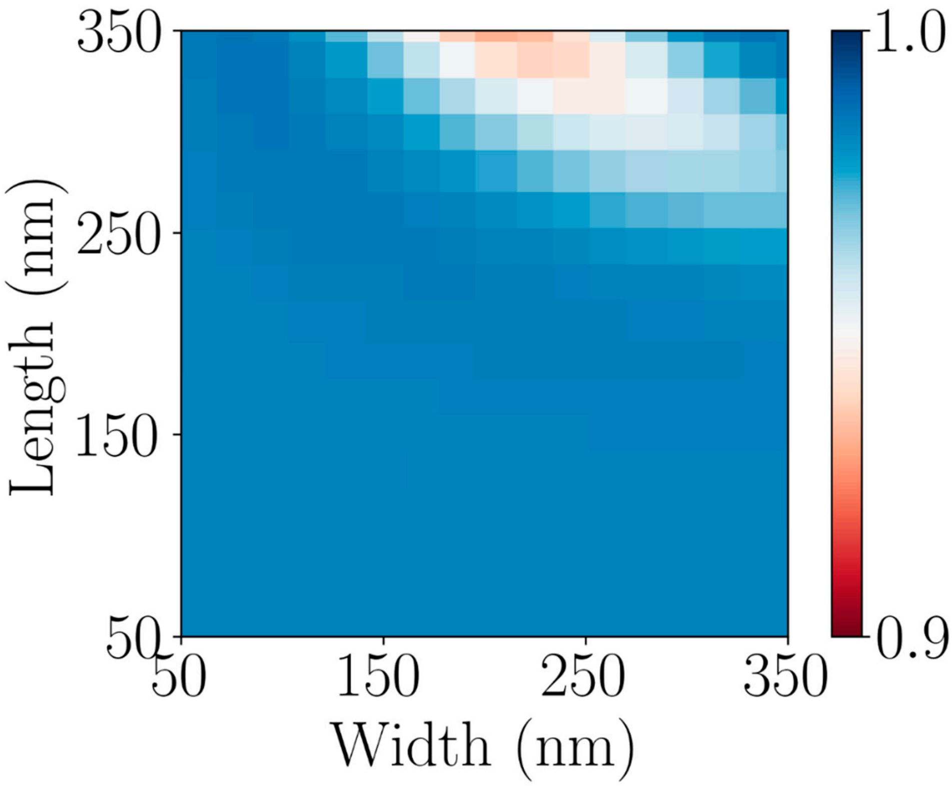

Figure S4. The amplitude of the complex transmission coefficient of the nanopost.

Figure S4 displays the amplitude of the complex transmission coefficient for the nanopost. It is evident that the amplitude for all nanoposts within the sampled range exceeds 0.94.

S4. The Details about Inverse-Designed Meta-Atom

Table S2. Prediction and re-simulation result for inverse-designed meta-atom.

t_x: 0.5, t_y: 0.5			t_x: 0.5 − 0.5i, t_y: 0.5 − 0.5i		
**Prediction**	**Simulation**	**Error**	**Prediction**	**Simulation**	**Error**
t_x: 0.36 − 0.03i<br>t_y: 0.54 − 0.003i	t_x: 0.37 − 0.11i<br>t_y: 0.54 − 0.03i	e_x: 0.17<br>e_y: 0.05	t_x: 0.52 − 0.44i<br>t_y: 0.48 − 0.53i	t_x: 0.57 − 0.47i<br>t_y: 0.51 − 0.53i	e_x: 0.08<br>e_y: 0.03
t_x: 0.42 − 0.03i<br>t_y: 0.47 − 0.23i	t_x: 0.41 − 0.06i<br>t_y: 0.47 − 0.2i	e_x: 0.11<br>e_y: 0.2	t_x: 0.50 − 0.46i<br>t_y: 0.49 − 0.44i	t_x: 0.53 − 0.47i<br>t_y: 0.54 − 0.46i	e_x: 0.04<br>e_y: 0.06
t_x: 0.46 − 0.18i<br>t_y: 0.51 − 0.19i	t_x: 0.45 − 0.16i<br>t_y: 0.59 − 0.19i	e_x: 0.17<br>e_y: 0.21	t_x: 0.53 − 0.43i<br>t_y: 0.48 − 0.51i	t_x: 0.57 − 0.46i<br>t_y: 0.53 − 0.52i	e_x: 0.08<br>e_y: 0.04
t_x: 0.43 − 0.02i<br>t_y: 0.47 − 0.26i	t_x: 0.43 − 0.02i<br>t_y: 0.48 − 0.23i	e_x: 0.07<br>e_y: 0.23	t_x: 0.53 − 0.43i<br>t_y: 0.48 − 0.50i	t_x: 0.57 − 0.46i<br>t_y: 0.53 − 0.52i	e_x: 0.08<br>e_y: 0.04
**t_x: 0.5, t_y: 0.5 − 0.5i**			**t_x: 0.5, t_y: −0.5i**		
**Prediction**	**Simulation**	**Error**	**Prediction**	**Simulation**	**Error**
t_x: 0.50 − 0.09i<br>t_y: 0.49 − 0.46i	t_x: 0.48 − 0.11i<br>t_y: 0.48 − 0.43i	e_x: 0.11<br>e_y: 0.07	t_x: 0.48 − 0.02i<br>t_y: −0.01 − 0.42i	t_x: 0.51 − 0.06i<br>t_y: −0.02 − 0.49i	e_x: 0.06<br>e_y: 0.02
t_x: 0.47 − 0.09i<br>t_y: 0.48 − 0.46i	t_x: 0.46 − 0.06i<br>t_y: 0.48 − 0.42i	e_x: 0.07<br>e_y: 0.08	t_x: 0.48 − 0.01i<br>t_y: 0.08 − 0.49i	t_x: 0.45 − 0.11i<br>t_y: 0.03 − 0.49i	e_x: 0.12<br>e_y: 0.03
t_x: 0.44 − 0.06i<br>t_y: 0.57 − 0.50i	t_x: 0.48 − 0.06i<br>t_y: 0.54 − 0.50i	e_x: 0.06<br>e_y: 0.04	t_x: 0.48 − 0.06i<br>t_y: −0.06 − 0.54i	t_x: 0.45 − 0.15i<br>t_y: −0.05 − 0.58i	e_x: 0.16<br>e_y: 0.09
t_x: 0.44 − 0.08i<br>t_y: 0.46 − 0.45i	t_x: 0.44 − 0.09i<br>t_y: 0.45 − 0.42i	e_x: 0.11<br>e_y: 0.09	t_x: 0.45 − 0.09i<br>t_y: −0.01 − 0.50i	t_x: 0.42 − 0.14i<br>t_y: −0.06 − 0.51i	e_x: 0.16<br>e_y: 0.06

S5. Comparison with Phase-Only Metasurface

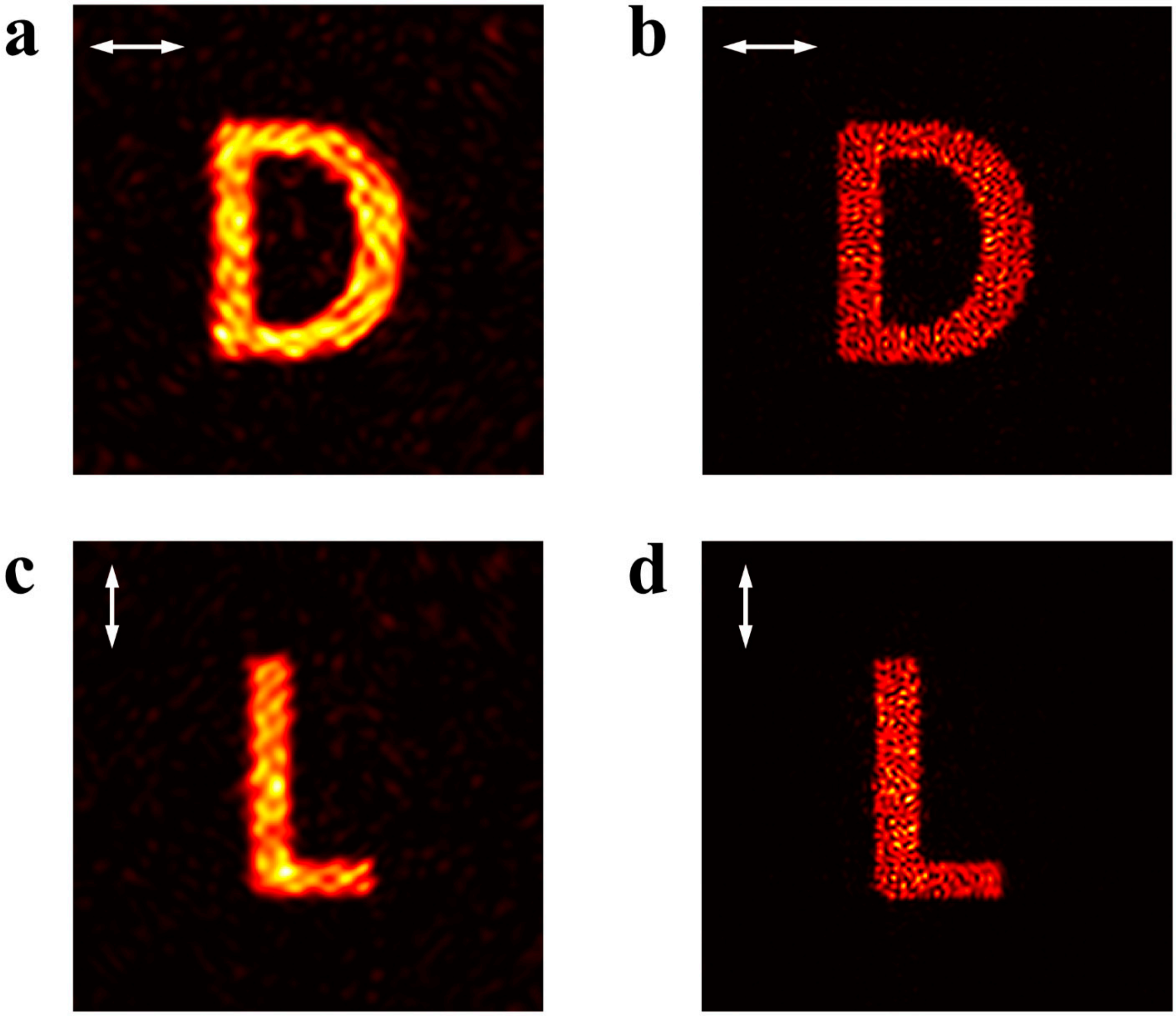

Figure S5. Comparison between a) and c) complex amplitude and b) and d) phase-only metasurface holography.

In this study, we have designed complex amplitude metasurfaces capable of generating high quality holographic images, in contrast to phase-only metasurfaces. Figure S3 provides a comparison between these two metasurface types. Specifically, Figure S3a and c correspond to the two lower images in Figure 5d, which are holographic images created by the INN-retrieved complex amplitude metasurface. Figures S3b and d display the images produced by the phase-only hologram. For the phase-only hologram, we obtained the target phase distribution for both x- and y-polarization using the Gerchberg-Saxton (GS) algorithm. We then employed a brute-force method within the training dataset to identify an appropriate complex meta-atom for each pixel. To ensure a more equitable comparison between these two methods, we maintained the use of the complex meta-atom as the fundamental building block for the metasurface obtained through the GS algorithm. The goal was to minimize the mean square error (MSE) between the target phase delay at each pixel, as determined by the GS algorithm, and the complex transmission coefficient achievable through the meta-atom. It is evident that the holographic images created by the metasurface retrieved using the INN method exhibit superior quality and a higher signal-to-noise ratio when compared to the speckle images generated by the metasurface retrieved through the GS algorithm.

S6. Training INN with Fewer Samples

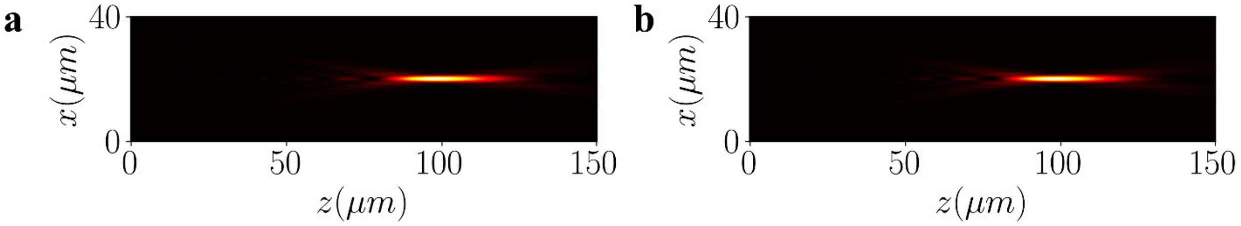

Figure S6. The inverse designed metalens using INN trained with a) 9,000 data points and b) 7,000 data points.

The INN model can achieve effective training with a reduced number of samples while maintaining satisfactory performance. Figure S6 displays the inverse-designed metalens outcomes using INN trained with 9,000 data points and 7,000 data points, respectively, and they demonstrate comparable performance.

References

1. Szegedy, C.; Ioffe, S.; Vanhoucke, V.; Alemi, A. In *Inception-v4, inception-resnet and the impact of residual connections on learning*, Proceedings of the AAAI conference on artificial intelligence, 2017.
2. Ardizzone, L.; Kruse, J.; Wirkert, S.; Rahner, D.; Pellegrini, E. W.; Klessen, R. S.; Maier-Hein, L.; Rother, C.; Köthe, U., Analyzing inverse problems with invertible neural networks. *arXiv preprint arXiv:1808.04730* **2018**.
3. Gretton, A.; Borgwardt, K. M.; Rasch, M. J.; Schölkopf, B.; Smola, A., A kernel two-sample test. *The Journal of Machine Learning Research* **2012,** *13* (1), 723-773.

Disclaimer/Publisher's Note: The statements, opinions and data contained in all publications are solely those of the individual author(s) and contributor(s) and not of MDPI and/or the editor(s). MDPI and/or the editor(s) disclaim responsibility for any injury to people or property resulting from any ideas, methods, instructions or products referred to in the content.
